# Examining the effectiveness of a deep learning-based computer-aided breast cancer detection system for breast ultrasound

**DOI:** 10.1007/s10396-023-01332-9

**Published:** 2023-07-04

**Authors:** Tomoyuki Fujioka, Kazunori Kubota, Jen Feng Hsu, Ruey Feng Chang, Terumasa Sawada, Yoshimi Ide, Kanae Taruno, Meishi Hankyo, Tomoko Kurita, Seigo Nakamura, Ukihide Tateishi, Hiroyuki Takei

**Affiliations:** 1 Department of Diagnostic Radiology, Tokyo Medical and Dental University, 1-5-45 Yushima, Bunkyo-ku, Tokyo, 113-8510 Japan; 2 Department of Radiology, Dokkyo Medical University Saitama Medical Center, 2-1-50 Minami-Koshigaya, Koshigaya, Saitama 343-8555 Japan; 3 Department of Computer Science and Information Engineering, National Taiwan University, No. 1, Sec. 4, Roosevelt Rd, Taipei, 10617 Taiwan, ROC; 4 Department of Breast Surgery, NTT Medical Center Tokyo, 5-9-22 Higashi-Gotanda, Shinagawa-ku, Tokyo, 141-8625 Japan; 5 Department of Breast Surgical Oncology, Department of Surgery, Showa University School of Medicine, 1-5-8 Hatanodai, Shinagawa-ku, Tokyo, 142-8666 Japan; 6 Department of Breast Oncology, Kikuna Memorial Hospital, 4-4-27 Kikuna, Kohoku-ku, Yokohama, 222-0011 Japan; 7 Department of Breast Surgical Oncology, Nippon Medical School, 1-1-5 Sendagi, Bunkyo-ku, Tokyo, 113-8602 Japan

**Keywords:** Breast cancer, Ultrasound, Deep learning, Artificial intelligence, Computer-aided detection

## Abstract

**Purpose:**

This study aimed to evaluate the clinical usefulness of a deep learning-based computer-aided detection (CADe) system for breast ultrasound.

**Methods:**

The set of 88 training images was expanded to 14,000 positive images and 50,000 negative images. The CADe system was trained to detect lesions in real- time using deep learning with an improved model of YOLOv3-tiny. Eighteen readers evaluated 52 test image sets with and without CADe. Jackknife alternative free-response receiver operating characteristic analysis was used to estimate the effectiveness of this system in improving lesion detection.

**Result:**

The area under the curve (AUC) for image sets was 0.7726 with CADe and 0.6304 without CADe, with a 0.1422 difference, indicating that with CADe was significantly higher than that without CADe (*p* < 0.0001). The sensitivity per case was higher with CADe (95.4%) than without CADe (83.7%). The specificity of suspected breast cancer cases with CADe (86.6%) was higher than that without CADe (65.7%). The number of false positives per case (FPC) was lower with CADe (0.22) than without CADe (0.43).

**Conclusion:**

The use of a deep learning-based CADe system for breast ultrasound by readers significantly improved their reading ability. This system is expected to contribute to highly accurate breast cancer screening and diagnosis.

## Introduction

Breast cancer is the most common cancer in females and is the second leading cause of cancer-related death [1]. Mammography is the most widely used modality, with screening mammography reducing breast cancer mortality. However, patients with dense breasts have a low breast cancer detection rate on mammography [2]. Additionally, mammography has the disadvantages of radiation exposure and pain. Ultrasound imaging is a widely used modality for breast cancer detection and diagnosis when abnormalities are found in other imaging modalities such as mammography or clinical examination [3, 4]. In recent years, the breast cancer detection rate was proven to increase when mammography and ultrasound are used together, and ultrasound is expected to play an important role as a breast cancer screening modality [5, 6]. However, problems associated with ultrasound are its dependence on the technique of the operator, a lack of experienced readers, and poor reproducibility.

In recent years, research in artificial intelligence (AI), especially deep learning methods, has advanced and been applied to medical imaging [7–10]. Additionally, its use has been extended to breast imaging, and several studies have investigated its application for image classification, object detection, segmentation, and generative imaging of breast tumors [11–17]. A Food and Drug Administration (FDA)-approved deep learning-based computer-aided detection (CADe) system for breast ultrasound exists in the United States and is used in clinical practice [18]. Conversely, Japan has no Pharmaceuticals and Medical Devices Agency (PMDA)-approved breast cancer detection support system for ultrasound in clinical practice; thus, developing such a system and evaluating its clinical utility are required.

We obtained a deep learning-based CADe system for use in the present study: a breast free-hand ultrasound (BR-FHUS) Smart System (Taihao Medical Inc., Taipei City, Taiwan) for breast ultrasound developed in Taiwan. This system has been evaluated worldwide, but its performance is unclear in Japan. Evaluating the usefulness in the country where it will be used is necessary when introducing a new system from abroad. Therefore, this study aimed to evaluate the clinical usefulness of a deep learning-based CADe system for breast ultrasound in Japan. Based on the study results, this CADe system was approved by the PMDA for the first time in Japan (Approval date: November 24, 2020; Approval number: 30200BZX00379000).

## Materials and methods

### Patients

Informed consent for participation in this study and the use of ultrasound images was obtained at one institution in Taiwan where the images were recorded. This study does not fall under the ethical guidelines for medical research involving human subjects and compliance with the Declaration of Helsinki because this study was designed to evaluate the performance of this system using only non-personally identifiable ultrasound image data.

## Data set

The data used in this study were from patients who visited the department of breast surgery at one institution in Taiwan between November 2014 and April 2015. Eighty-eight image sets from 45 cases were used as training images, and 232 image sets from 232 cases were used as validation images. Images were acquired on a Voluson E6 (GE Healthcare, Illinois, USA) or ACUSON S200 (Siemens Healthcare, Erlangen, Germany). One breast case was considered one image set; one image set contained a movie consisting of approximately 700 B-mode images.

## Development of a deep learning-based CADe system

Breast lesions suspected of being breast cancer in the 88 training image sets were labeled by breast surgeons at one institution in Taiwan based on ultrasound images and clinical information. The training image sets were expanded to 14,000 positive images and 50,000 negative images by data augmentation with rotation, shift, and horizontal transformation.

**Fig. 1:**
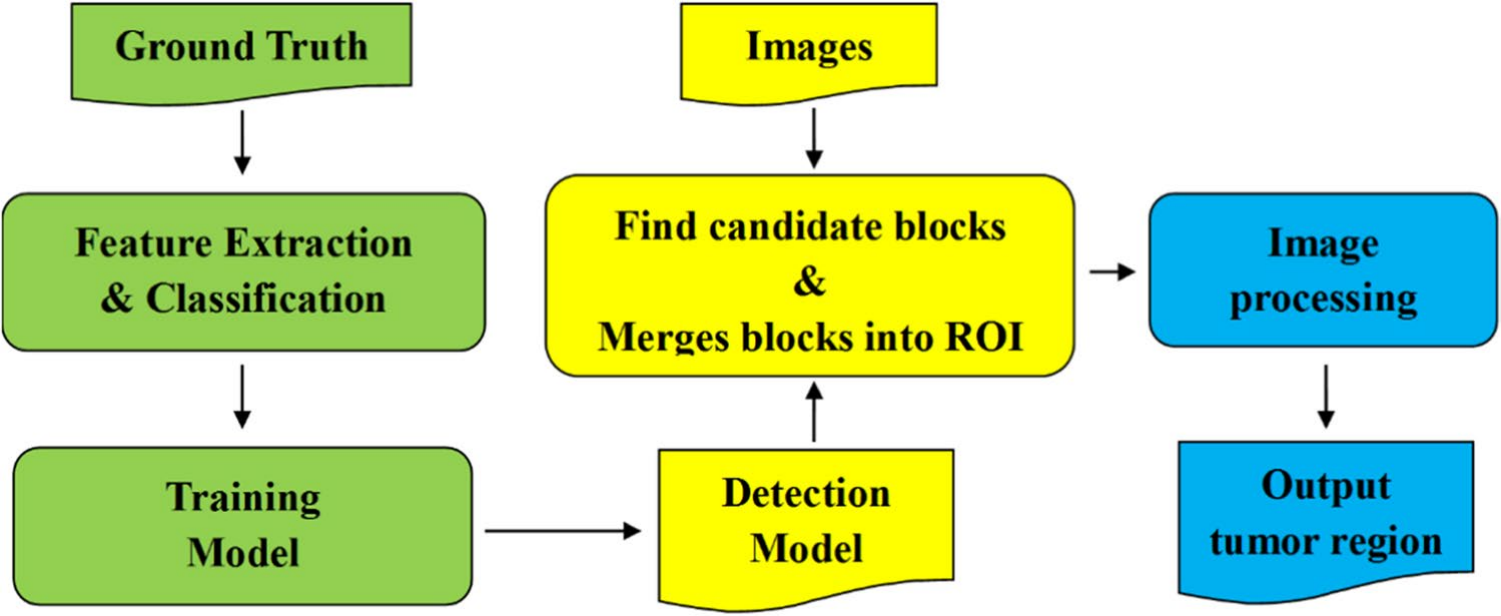
Flowchart of the whole process, The proposed method extracted Haar-like features (based on intensity and shape) from ultrasound images and used the cascade classifier to select the features for the pre-training model. Further, the transfer learning method was used to initialize the pre-training weights from the cascade classifier and then train a detection model for finding the regions of the lesion in real-time

**Fig. 2:**
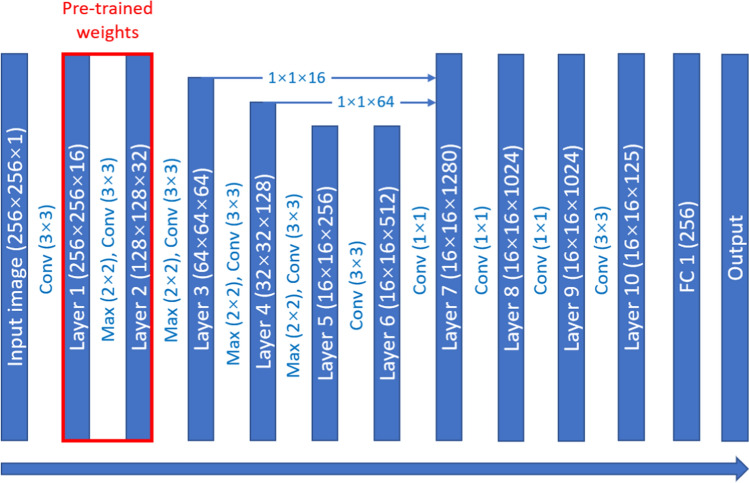
Architecture of modified YOLOv3-tiny, The transfer learning method was used to initialize the weights of the pre-training model of deep learning. The deep learning model used in the study was a modified YOLOv3-tiny model. Using a YOLOv3-tiny architecture is ideal since the network is relatively shallow to YOLOv3 architecture and suitable for real-time detection

Figure 1 shows the flowchart of the whole process of real-time computer-assisted lesion detection. The proposed method extracted Haar-like features (based on intensity and shape) from ultrasound images and used the cascade classifier to select the features for the pre-training model. Further, the transfer learning method was used to initialize the pre-training weights from the cascade classifier and then train a detection model for real-time findings in the regions of the lesion. The detailed process steps are as follows:

- Step 1.Extract the Haar-like features from the ground truth images.
- Step 2.Use a cascade classifier to select the features for the pre-training model.
- Step 3.Use the transfer learning method to initialize the weights of the pre-training model of deep learning. The deep learning model used in the study was the modified YOLOv3-tiny model shown in Fig. 2. Using a YOLOv3-tiny architecture is ideal since the network is relatively shallow to the YOLOv3 architecture and suitable for real-time detection [19].
- Step 4.Train the detection model using ground truth images and pre-training weights.
- Step 5.Find the candidate blocks from ultrasound images and merge the blocks into the region of interest (ROI).
- Step 6.Use the image processing method (edge detection and mathematical morphology) to eliminate the wrong (non-lesion) regions.
- Step 7.Finally, output the lesion regions and then compare them with the ground truth for clock and distance. A difference within 2 cm indicates that the region hit the ground truth.

## Description of the CADe system

**Fig. 3:**
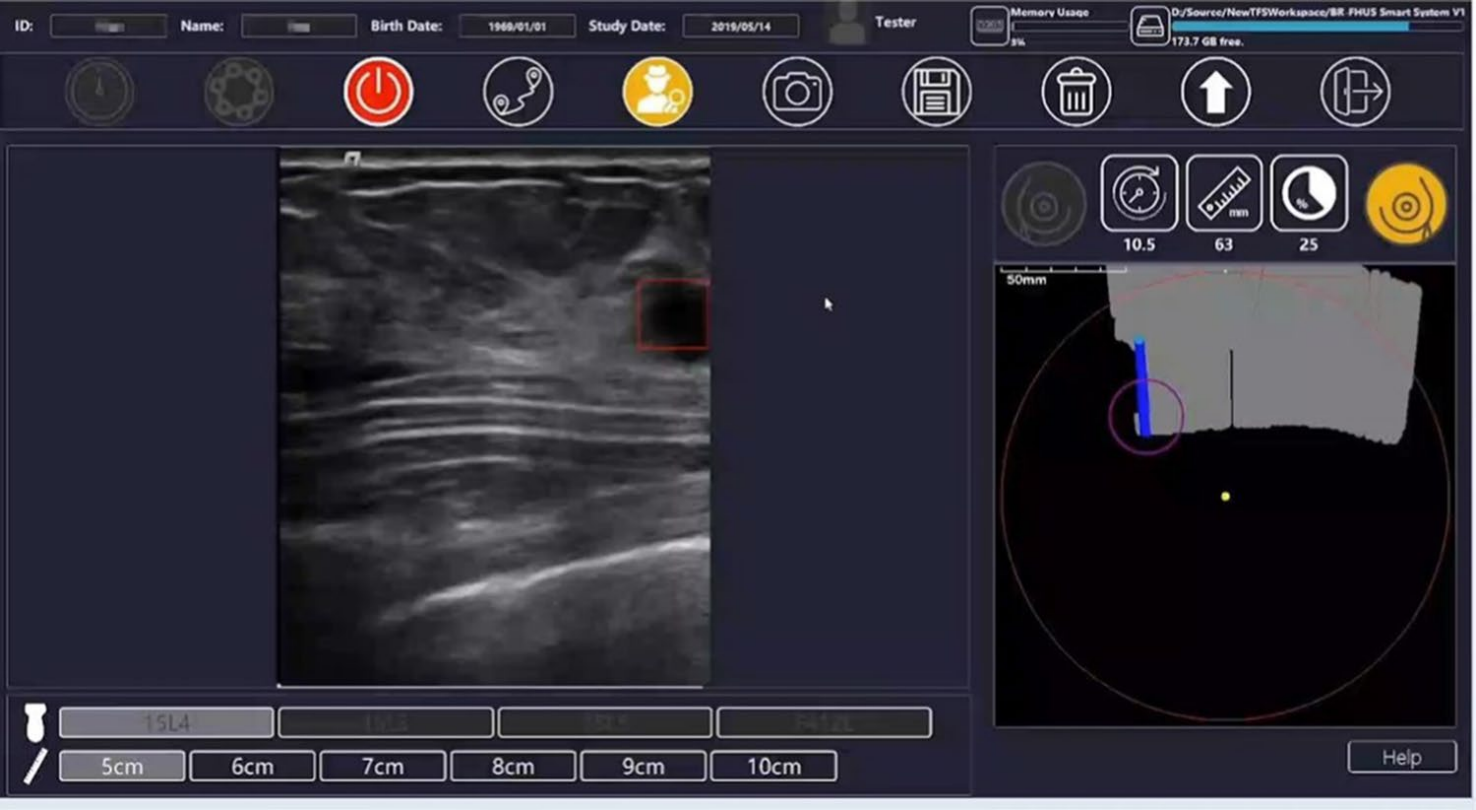
Route map and region of suspected breast cancer displayed by the CADe system, The CADe system; breast free-hand ultrasound (BR-FHUS). The BR-FHUS Smart System is based on AI technology and has several functions. It displays the trajectory of the probe of the ultrasound imaging system as a route map, performs shape analysis during the examination, and immediately visualizes areas suspected of having breast cancer as ROIs on the image screen

The CADe system, BR-FHUS Smart System, is based on artificial intelligence (AI) technology and has the following functions:

1. It displays the trajectory of the probe of the ultrasound imaging system as a route map. It alerts the user when there is a risk of scan omissions such as high movement speed.
2. It performs shape analysis during the examination and immediately displays areas suspected of having breast cancer as ROIs on the image screen. The probe position at that time is displayed on the route map screen (Fig. 3).

## Definition of the standard of truth (SOT)

The SOT for the region of suspected breast cancer for the exam was prepared as follows:

1. The SOT was determined by three experts with > 10 years of experience in reading breast ultrasound.
  - The order of the image sets was randomized, and the image sets were distributed to the three experts so that two experts per case could independently evaluate each image set. They evaluated the images with reference to the guidelines for breast ultrasound published by the Japan Association of Breast and Thyroid Sonology (JABTS) [20].
  - The order of the image sets was randomized, and the image sets were distributed to the three experts so that two experts per case could independently evaluate each image set. They evaluated the images with reference to the guidelines for breast ultrasound published by the Japan Association of Breast and Thyroid Sonology (JABTS) [20].
2. The expert then referred to the images evaluated by the other two experts to determine the SOT.
  - If only one expert marked an image, the mark was selected for adoption or rejection.
  - If two experts marked an image, the one marked by an expert to be adopted was selected.
  - The images marked by two or more experts were designated as the SOT.

Excluding two from the 232 test image sets, 230 SOTs were determined. The number of lesions per case, lesion size per case, and number of categories of cases are shown in Table 1.

**Table 1:** Breast disease pathology in test data

Breast disease name	Number of cases
Invasive ductal carcinoma	57
Ductal carcinoma in situ	11
Invasive lobular carcinoma	2
Invasive micropapillary carcinoma	2
Malignancy others	8
Fibroadenoma	14
Fibroepithelial lesion	5
Fibrocystic change	10
Ductal epithelial cells	9
Benign others	15
No biopsy	89
No record	10
Total	232

## Stand-alone performance test of the CADe

**Fig. 4:**
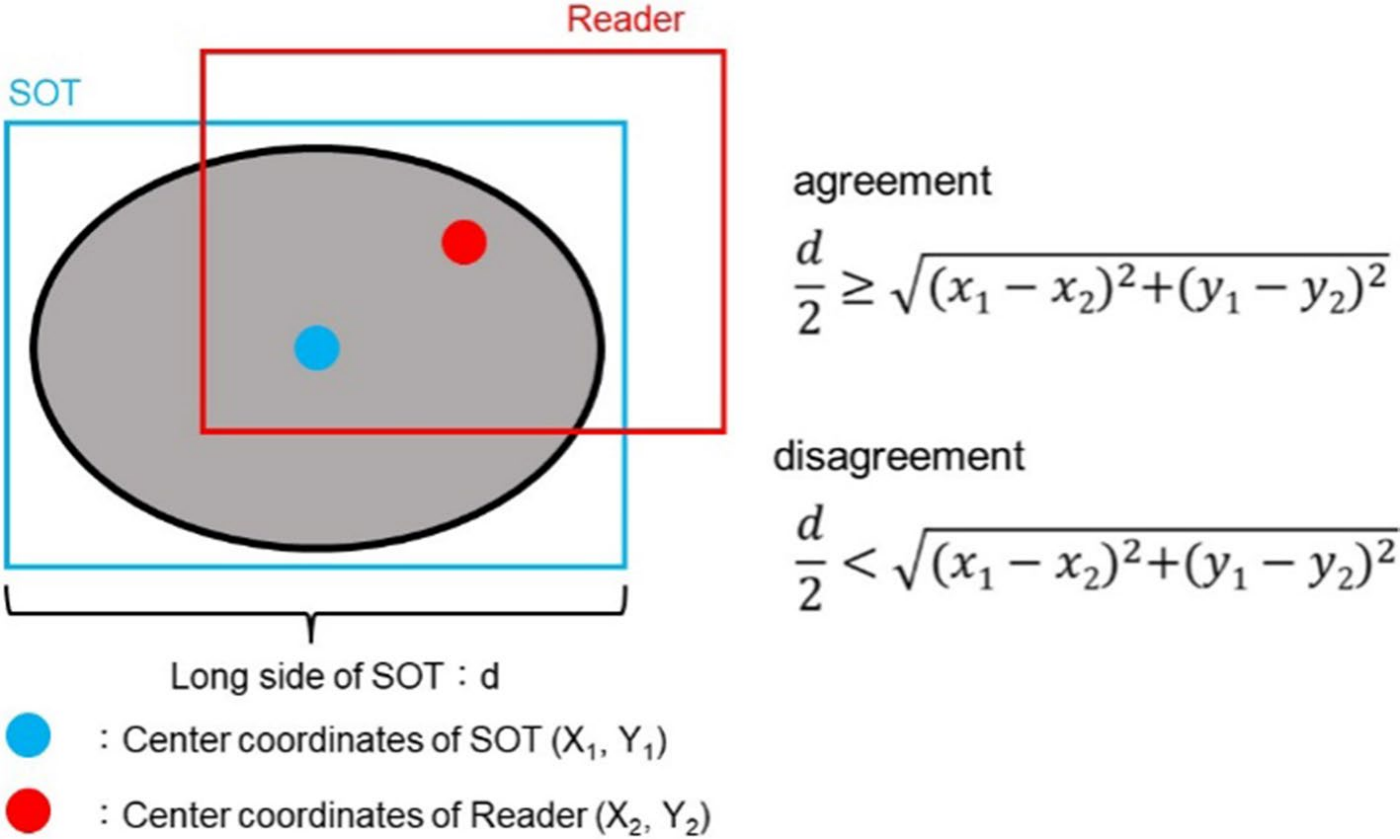
Agreement or disagreement between the SOT and the reader’s result, Agreement or disagreement between the SOT and the readers’ results per frame image was determined by the position of the center coordinates of the SOT and the reader’s ROI and the long edge of the SOT

The 230 test image sets labeled as SOTs were used for the stand-alone performance test of the CADe. The results for CADe were compared with the SOT, and agreement or disagreement was determined. The agreement or disagreement between the SOT and the readers’ results per frame image was determined by the position of the center coordinates of the SOT and the reader’s ROI and the long edge of the SOT, as shown in Fig. 4.

## Image evaluations by multiple readers with and without CADe

**Fig. 5:**
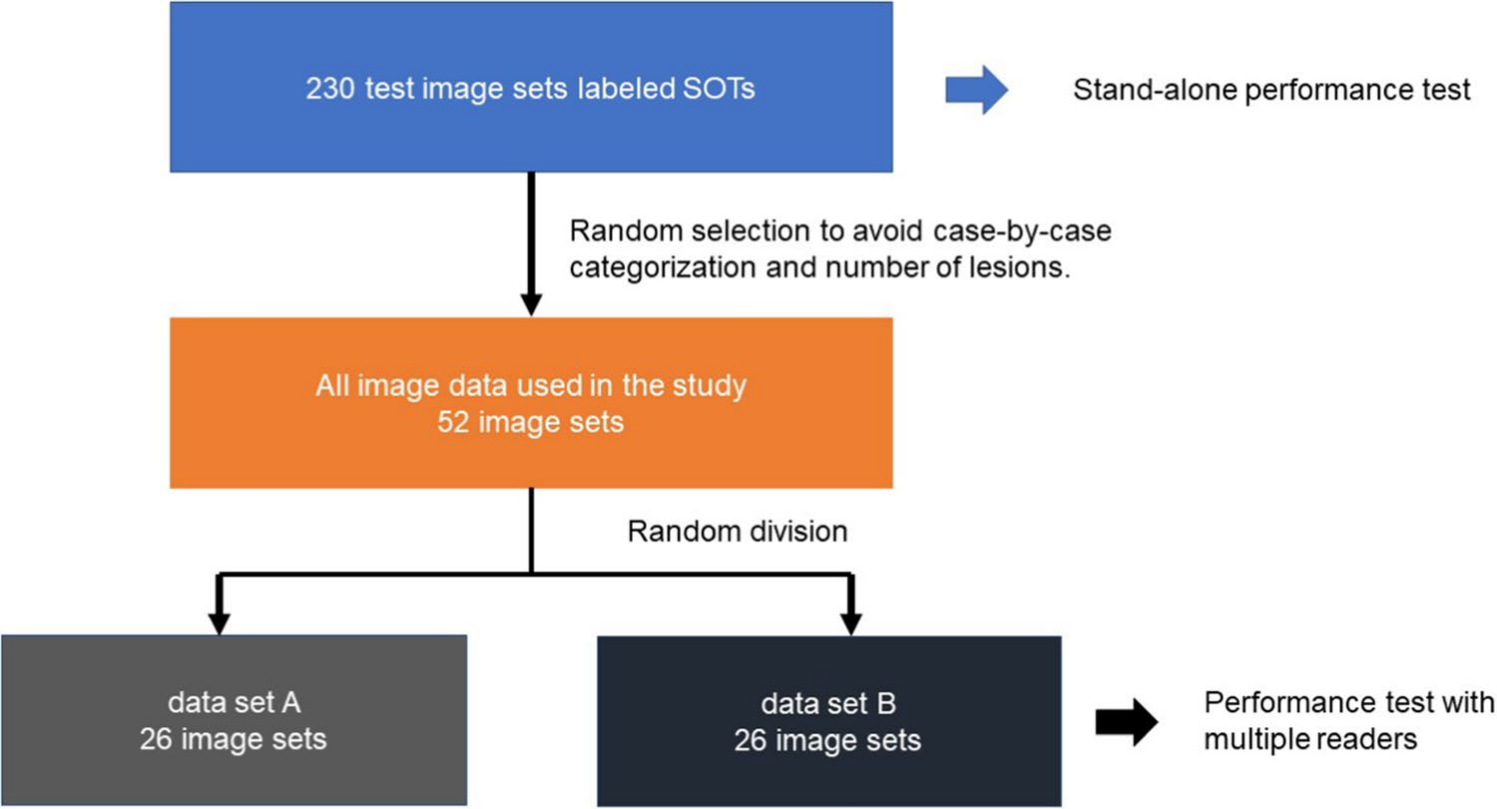
Assignment of test image sets for performance testing of the CADe system by multiple readers, **A** set of 230 test images labeled as SOT was used for the stand-alone performance test of CADe. Of them, 52 image sets were randomly selected to avoid bias in the case-by-case categorization and number of lesions. Additionally, these image sets were randomly split into two image data sets, i.e., image data sets **A** and **B**, which were used for performance testing by multiple readers

From the 230 test image sets labeled as SOTs, 52 image sets were randomly selected to avoid bias in the case-by-case categorization and number of lesions. Furthermore, these image sets were randomly divided into two sets, image data sets A and B. The preparation flow of the evaluated image data is shown in Fig. 5. Image evaluations by multiple readers were performed as follows:

The readers included six specialists with > 5 years of experience in reading breast ultrasound (three breast surgeons and three radiologists), six residents with < 5 years of experience (three breast surgeons and three radiologists), and six well-trained clinical technologists with > 3 years of experience. A break of at least 10 min per hour of reading was provided, considering reader fatigue, but the break time was not included in the reading time. The readers evaluated the test image sets on video. They were allowed to manipulate the frames of the video freely.

1. The reading study was a crossover pre- and post-intervention comparison study consisting of two sessions.
  1. Team X read image data set A without CADe and image data set B with CADe, and Team Y read image data set A with CADe and image data set B without CADe.
  2. Team X read image data set A without CADe and image data set B with CADe, and Team Y read image data set A with CADe and image data set B without CADe.
  3. was started > 4 weeks after the completion of Session 1.
2. The readers read the image data in each session and extracted the frame images that were suspected of having breast cancer. However, a maximum of three frame images were extracted for the same lesion in the order in which they were considered to have high serial confidence.
3. The reader created an ROI in the extracted frame images by enclosing the border of the suspected breast cancer lesion and the surrounding adjacent area in a rectangle. An ROI that could not be created, because a suspected breast cancer lesion was not observed, was recorded.
4. For each ROI, the reader evaluated the serial confidence level of the suspected breast cancer site. Continuous confidence was evaluated using a 100-mm straight line that considered the categorization and was recorded on the Visual Analogue Scale form. The same lesion number was assigned to the ROI for the same lesion for each ROI, and the lesion linkage was recorded.

The results of each reader with and without CADe were compared with the SOT, and agreement or disagreement was determined (Fig. 4).

## Statistical analysis

Jackknife alternative free-response receiver operating characteristic (JAFROC) analysis was used to estimate the effectiveness of this system in improving lesion detection [21]. JAFROC analysis by reader group (specialists, residents, and clinical technologists) was also performed. A *P*-value of < 0.05 was considered statistically significant.

The sensitivity per lesion suspected of being breast cancer, as well as the sensitivity, specificity, positive predictive value (PPV), negative predictive value (NPV), and false positive per case (FPC) per case (breast), were calculated. The time required for reading was then calculated. The lesions that were not detected without CADe but were detected with CADe and the lesions that were not detected with CADe but were detected without CADe were tabulated.

## Results

The results of the stand-alone performance test of CADe showed a sensitivity per lesion of 95.5% (95% confidence interval [CI] 90.1–96.9%), a sensitivity per case of 100% (95% CI 100–100%), a specificity of 2.2% (95% CI 0–5.1%), and a mean number of false positives per case of 28.1 (95% CI 27.5–28.8%).

During the performance test by multiple readers, one image set was found to contain image data from other patients; thus, this image set was excluded from the analysis. Ultimately, 51 image sets were analyzed in this study.

Table 2 shows the JAFROC analysis results. For all readers, the area under the curve (AUC) of the ROC curve with CADe was 0.7726 (95% CI 0.6993–0.8459), and that without CADe was 0.6304 (95% CI 0.5240–0.7368). The difference in AUCs of ROC curves with and without CADe was 0.1422 (95%CI 0.0808–0.2036); thus, it was significantly higher with CADe than without CADe (*p* < 0.0001). Subgroup analysis by specialists, residents, and clinical technologists revealed that the AUC with CADe was significantly higher than that without CADe (*p* = 0.0157–0.0207).

**Table 2:** Jackknife alternative free-response receiver operating characteristic analysis

Readers	With or without CADe	AUC	95% IC	*P*-value
All (*n* = 18)	With CADe	0.7726	0.6993–0.8459	
	Without CADe	0.6304	0.5240–0.7368	
	(With CADe) – (Without CADe)	(0.1420)	0.0808–0.2036	< 0.0001
Specialists (*n* = 6)	With CADe	0.7430	0.6528–0.8332	
	Without CADe	0.6245	0.4963–0.7527	
	(With CADe) – (Without CADe)	(0.1185)	0.0282–0.2089	0.0157
Residents (*n* = 6)	With CADe	0.7706	0.6725–0.8687	
	Without CADe	0.5837	0.4701–0.6974	
	(With CADe) – (Without CADe)	(0.1869)	0.0669–0.3068	0.0072
Clinical technologists (*n* = 6)	With CADe	0.8042	0.7152–0.8932	
	Without CADe	0.6830	0.5914–0.7745	
	(With CADe) – (Without CADe)	0.1213	0.0278–0.2147	0.0207

*AUC* area under the curve, *CI* confidence interval, *CADe* computer-assisted detection

The sensitivity per lesion for suspected breast cancer, as well as sensitivity, specificity, PPV, NPV, and FPC per case (breast), are shown in Table 3. The sensitivity per lesion was higher with CADe (61.3%) than without CADe (44.8%), and the sensitivity per case was higher with CADe (95.4%) than without CADe (83.7%). The same trend was observed for the PPV. The specificity of suspected breast cancer cases with CADe (86.6%) was higher than that without CADe (65.7%). The same trend was observed for the NPV. The FPC was lower with CADe (0.22) than without CADe (0.43).

**Table 3:** Diagnostic outcomes with or without CADe per lesion and per case

With or without CADe	Lesion/Case	Evaluation method	Result	95% CI
With CADe	Lesion	Sensitivity	61.3%	57.1%–65.5%
Without CADe	Lesion	Sensitivity	44.8%	40.6%–49.1%
With CADe	Case	Sensitivity	95.4%	93.1%–97.8%
Without CADe	Case	Sensitivity	83.7%	79.5%–87.8%
With CADe	Case	Specificity	86.6%	83.9%–89.3%
Without CADe	Case	Specificity	65.7%	61.9%–69.4%
With CADe	Case	PPV	78.1%	73.9%–82.3%
Without CADe	Case	PPV	54.9%	50.4%–59.5%
With CADe	Case	NPV	97.4%	96.1%–98.8%
Without CADe	Case	NPV	88.9%	86.0%–91.8%
With CADe	Case	FPC	0.22	96.1%–98.8%
Without CADe	Case	FPC	0.43	86.0%–91.8%

CI: confidence interval, CADe: computer-assisted detection, PPV: positive predictive value, NPV: negative predictive value, FPC: false positives per case

The mean ± standard deviation of the time required for reading per case was 153.8 ± 126.2 s with CADe and 147.0 ± 99.6 s without CADe.

The number of lesions that were successfully detected without CADe but not with CADe was 45 (5.0%), and the number of lesions that were not detected without CADe but were successfully detected by CADe was 131 (14.5%).

**Fig. 6:**
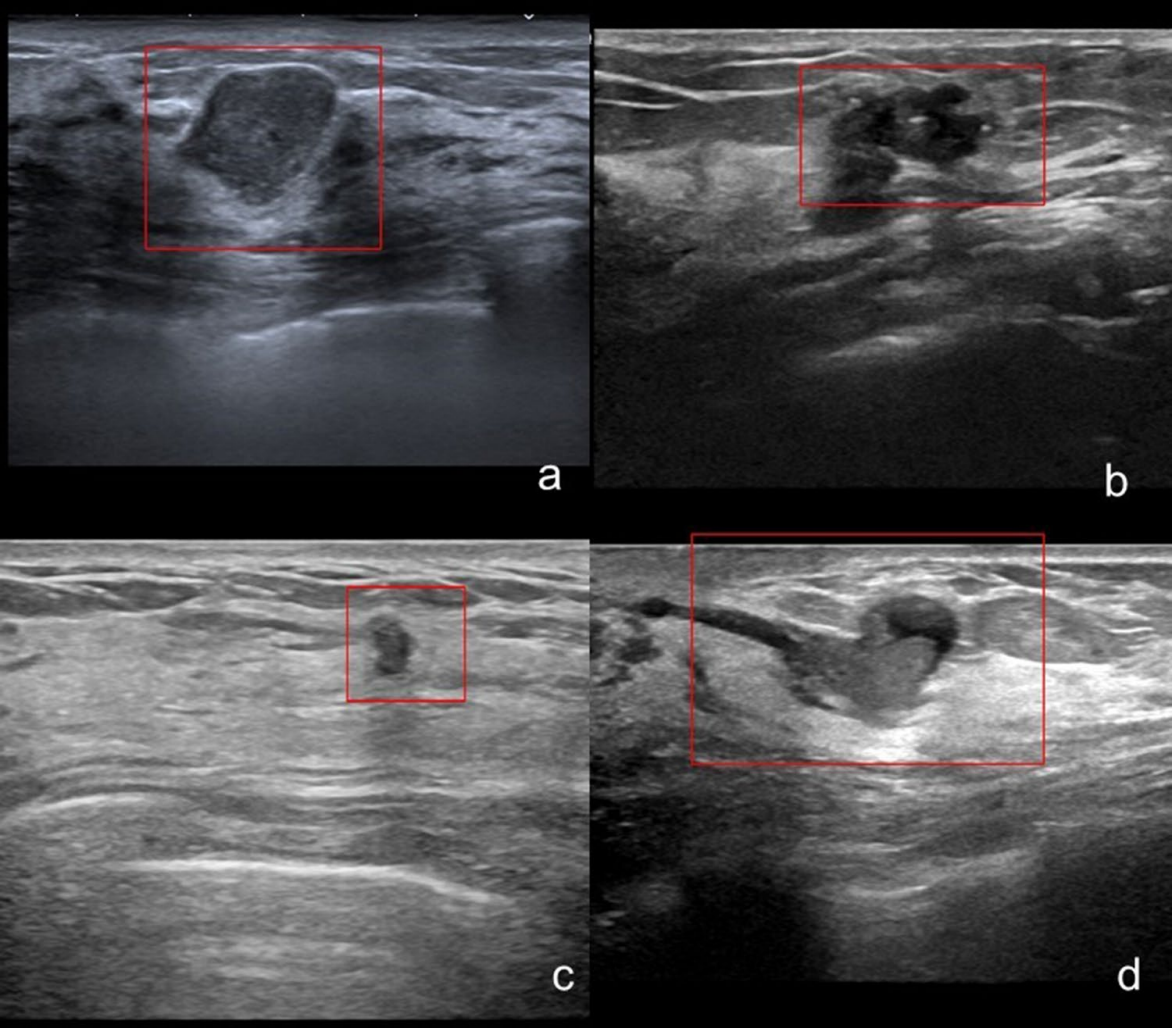
Representative true-positive cases, Representative true-positive cases are shown. The CADe system correctly detected lesions of various sizes and shapes (**a**–**d**)

**Fig.7:**
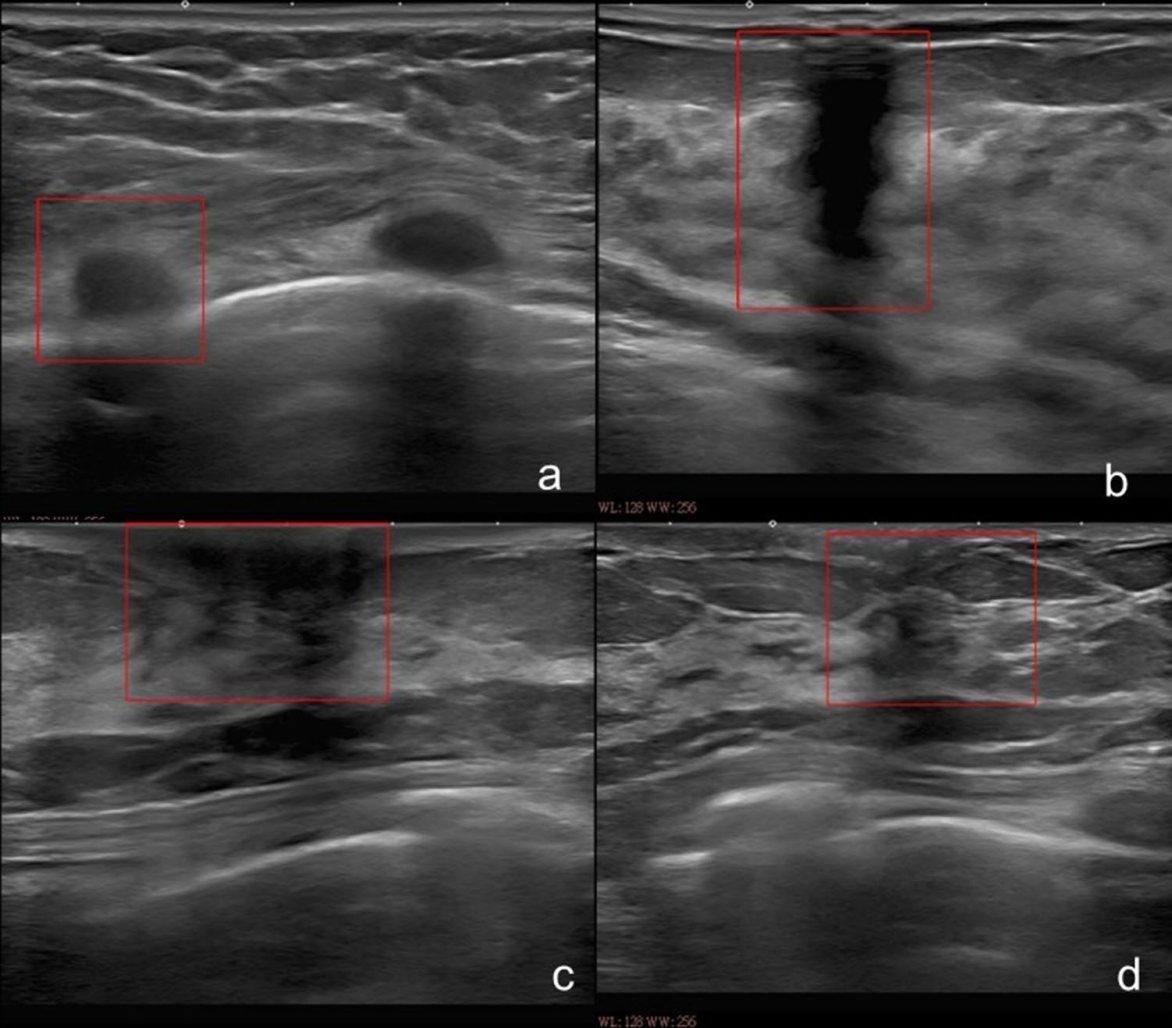
Representative false-positive cases, Representative false-positive cases are shown: the CADe system falsely detected a rib (**a**), a shadow (**b**), a nipple (**c**), and normal breast tissue (**d**)

Representative true-positive and false-positive cases are presented in Figs. 6, 7.

## Discussion

Over the past few years, advanced analytical methods such as texture analysis, radiomics, and deep learning have gained attention in the field of imaging research, and these advanced methods allow for more accurate and detailed analysis of the information contained in images compared to the traditional interpretation of image findings [8, 22–24].

This study investigated the effectiveness of a deep learning-based breast cancer detection support system through a crossover pre- and post-intervention comparative study of multiple readers. The results obtained from FROC analysis in this study showed that image sets with CADe showed significantly higher AUC values than those without CADe, indicating that the use of this system by readers significantly improved their reading ability. Therefore, this system was able to obtain PMDA approval.

The stand-alone performance test of CADe showed high sensitivity but low specificity and several false positives. A performance test by multiple readers revealed a higher sensitivity per lesion with CADe than without CADe. Additionally, the sensitivity, specificity, PPV, and NPV per case were higher with CADe than without CADe. FPC was lower with CADe than without CADe.

Therefore, the use of this system for diagnostic imaging would enable more accurate detection of lesions suspected to be breast cancer. Additionally, this system is expected to be clinically useful for screening purposes by reducing unnecessary detailed examinations and biopsies.

The reading time with and without CADe was 153.8 ± 126.2 s and 147.0 ± 99.6 s, respectively, showing a slight trend toward longer reading times with CADe. Although we estimate that this longer reading time with the use of CADe is not clinically problematic and is acceptable, further research is needed to prove how CADe affects the daily work of clinicians. This was the first time the readers evaluated ultrasound images with CADe; thus, they may have taken more time. They may perform more efficient readings when they become sufficiently familiar with the use of CADe. Additionally, reader stratification analysis suggested that reading performance was better with CADe than without CADe among specialists, residents, and clinical technologists. This indicates that CADe can potentially improve the performance of a wide range of readers, regardless of their years of experience or specialty.

Lesions can be detected more appropriately with CADe because 45 lesions (5.0%) changed from true positive to false negative while 131 lesions (14.5%) changed from false negative to true positive with CADe.

Several products in the United States have already received FDA approval and are used in actual clinical practice, although this system is the first CADe system for breast ultrasound using deep learning to be approved by PMDA [18]. In this study, we conducted a reading trial in Japan and proved the usefulness of this system developed in Taiwan. Sasaki et al. compared the breast cancer detection performance of a panel of three human readers and a stand-alone AI-based system, Transpara™ version 1.3.0 (ScreenPoint Medical BV, Nijmegen, The Netherlands), using digital mammograms in a population of 310 Japanese females. The AUC showed better diagnostic performance for the human reader than for the stand-alone Transpara system (human reader: 0.816, Transpara system: 0.706, difference: 0.11, *P* < 0.001). They found lower accuracy with the Transpara system than previous overseas results [25]. Verifying the usefulness of the system in the country where it will be used is necessary when implementing a system built using image data from another race or another country. Thus, we conducted a reading test in Japan using a model built in Taiwan with Taiwanese data. We may have been able to successfully evaluate the images because we are the same Asian people and our physiques are similar, but we need to accumulate data from the Japanese population to further evaluate the performance of this system in the future.

Lesions not included in the imaging area, small lesions, and mass-forming lesions may not be detected by the CADe system. Additionally, a rib, a shadow, a nipple, and normal mammary tissue appearing as a lesion may cause false positives on the CADe system. These false positives could be reduced by improving the AI to a more accurate model and training it with a variety of additional data. In addition, the observer should carefully check the original images to reduce missed lesions and unnecessary biopsies and follow-ups.

Readers evaluating the images were blinded to the patient’s data, but a diagnosis is based on patient information (physical examination, medical history, and family history) and findings from other modalities (mammography and magnetic resonance imaging) in practice. The physician should not only use the information from the CADe system but also combine multiple pieces of information to make a comprehensive diagnosis.

Research on CADe systems for breast ultrasound is ongoing, and some have already been approved in the United States, although we investigated a CADe system for breast ultrasound in this study. The CADe system has an even greater impact on human diagnosis; thus, it should be investigated more carefully when considering its application in Japan.

This system is only a detection aid, and the presence or absence of a lesion should not be determined based on the CADe system results alone. Stand-alone performance tests showed high sensitivity but low specificity and false positives. Only when human readers use this CADe system can its excellent performance be fully harnessed. Additionally, at present, the one accountable for the diagnosis if the AI makes a false diagnosis is unclear [26]. Compensation for patients and penalties for developers and medical professionals have not been determined. Thus, for the time being, using AI in clinical practice under the responsibility of medical professionals will be necessary.

This study had several limitations. First, this retrospective study had a relatively small number of cases, and it was validated using only a specific vendor’s ultrasound device. A larger prospective study with multiple devices from the United States is needed to further demonstrate the usefulness of this study. Second, the study was designed to focus on SOT detection as set by the experts. Investigation of the detection rate for malignancy and the PPV (malignancy rate) of the detected lesions with CADe readings is necessary.

## Conclusion

The use of a deep learning-based CADe system for breast ultrasound by readers significantly improved their reading ability. This system is expected to contribute to highly accurate breast cancer screening and diagnosis.
